# Application of hyperspectral technology in detection of agricultural products and food: A Review

**DOI:** 10.1002/fsn3.1852

**Published:** 2020-09-03

**Authors:** Min Zhu, Dan Huang, Xin‐Jun Hu, Wen‐Hua Tong, Bao‐Lin Han, Jian‐Ping Tian, Hui‐Bo Luo

**Affiliations:** ^1^ College of Bioengineering Sichuan University of Science and Engineering Zigong City Sichuan Province China; ^2^ College of Mechanical Engineering Sichuan University of Science and Engineering Zigong City Sichuan Province China; ^3^ Engineering Laboratory for Biological Brewing Technology of Bran Vinegar in the South of Sichuan Zigong China

**Keywords:** agricultural products and food, application, hyperspectral imaging technology, key points, quality and safety

## Abstract

Food is the foundation of human survival. With the development and progress of society, people increasingly focus on the problems of food quality and safety, which is closely related to human's health. Thus, the whole industrial chain from farmland to dining table need to be strictly controlled. Traditional detection methods are time‐consuming, laborious, and destructive. In recent years, hyperspectral technology has been more and more applied to food safety and quality detection, because the technology can achieve rapid and nondestructive detection of food, and the requirement to experimental condition is low; operability is strong. In this paper, hyperspectral imaging technology was briefly introduced, and its application in agricultural products and food detection in recent years was systematically summarized, and the key points in the research process were deeply discussed. This work lays a solid foundation for the peers to the following in‐depth research and application of this technology.

## Graphical abstract

1. Compared with chemical detection, the advantages of this technology were introduced.
2. The application progress of hyperspectral technology in primary agricultural products and food in recent years was systematically reviewed.
3. The deficiency and key points of this technology in the research were discussed in depth.

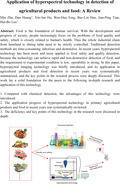

## 1 INTRODUCTION

China is the third largest country with land area in the world and is also a big agricultural country. Every year, the total amount of agricultural products is in the forefront of the world. Although the output of all kinds of food has increased, a problem that cannot be ignored has aroused people's attention, that is, food safety.

In particular, major food safety incidents in recent years, such as "red heart" duck eggs, “melamine” incident, and "lean meat powder" pork, once made China's food industry into a state of decline. The whole industrial chain from primary agricultural products to commodity was greatly affected. These problems not only appear in China, but also in other countries of the world. For example in 2005, the food standard agency of the UK found that salmon sold in the market contained a strong carcinogenic "malachite green," which shocked the world and caused panic.

As the saying goes, "food is the most important thing for the people," which also contains a meaning that food safety and quality is greater than heaven, because it is closely related to the health of human. The emergence of food safety incidents poses new challenge to traditional detection methods of food quality and safety, including the detection of the whole industrial chain from farmland to dining table. Traditional methods have great limitations, such as long detection cycle, strong destructive, complex operation, and single‐point detection. Consequently, spectral technology has gradually been introduced into food quality and safety detection, such as hyperspectral technology (Dale et al., 2013), infrared spectrum technology (Fu & Ying, 2016; Shi et al., 2012), and Raman spectrum technology (Ai et al., 2018). Among them, hyperspectral technology has unique advantage and has been widely used in astronomy (Hege, O'Connell, Johnson, Basty, & Dereniak, 2004), food (Feng & Sun, 2012; Gowen, O'Donnell, Cullen, Downey, & Frias, 2007), forensic examination (Edelman, Gaston, Van Leeuwen, Cullen, & Aalders, 2012; Malkoff & Oliver, 2000), crime scene investigation (Kuula et al., 2012; Schuler, Kish, & Plese, 2012), cultural relics protection (Fischer & Kakoulli, 2013; Liang, 2012), medicine (Afromowitz, Callis, Heimbach, DeSoto, & Norton, 1988; Carrasco, Gomez, Chainani, & Roper, 2003), plant and water conservation (Adam, Mutanga, & Rugege, 2010; Govender, Chetty, & Bulcock, 2007), and remote sensing mapping (Ren, Zabalza, Marshall, & Zheng, 2014). This technology not only can carry out large‐scale and rapid detection of objects, but also can retain the integrity to the greatest extent, which is an effective tool in the field of food nondestructive testing. Hyperspectral imaging technology is a image data technology with continuous narrow band, which can simultaneously detect the spectral and spatial information of objects, thus obtain more effective data. However, a obvious shortcoming of this technology lies in the high requirement for software and hardware, yet in recent years, with the enhancement of technological strength, the “short board” is also slowly being overcome.

In this paper, the principle and analysis process of hyperspectral imaging technology were briefly introduced, and the application of this technology in the field of agricultural products detection in recent years, including grains, fruits, vegetables, and meats, was summarized systematically. In addition, the deficiencies and key points in the research were discussed in depth. This work lays a solid foundation for the peers to the following in‐depth research and application of this technology.

## 2 HYPERSPECTRAL IMAGING TECHNOLOGY

As an emerging, nondestructive, and advanced optical technology, it is an image data technology with many narrow bands. It combines mechanical vision with spectral technology to detect the two‐dimensional spatial and one‐dimensional spectral information of the targets; thus, high‐resolution image and spectral data are obtained.

Therefore, the emergence of hyperspectral technology makes it easier to detect objects that cannot be detected with wide band. Moreover, compared with other optical technologies, hyperspectral image is closer to the real properties of objects. At present, this technology has developed rapidly, which can be divided into reflection imaging (Nicolaï, Lötze, Peirs, Scheerlinck, & Theron, 2006), fluorescence imaging (Vargas et al., 2010), and transmission imaging (Casasent, 2011). Among them, reflective imaging technology is the most commonly used.

**FIGURE 1:**
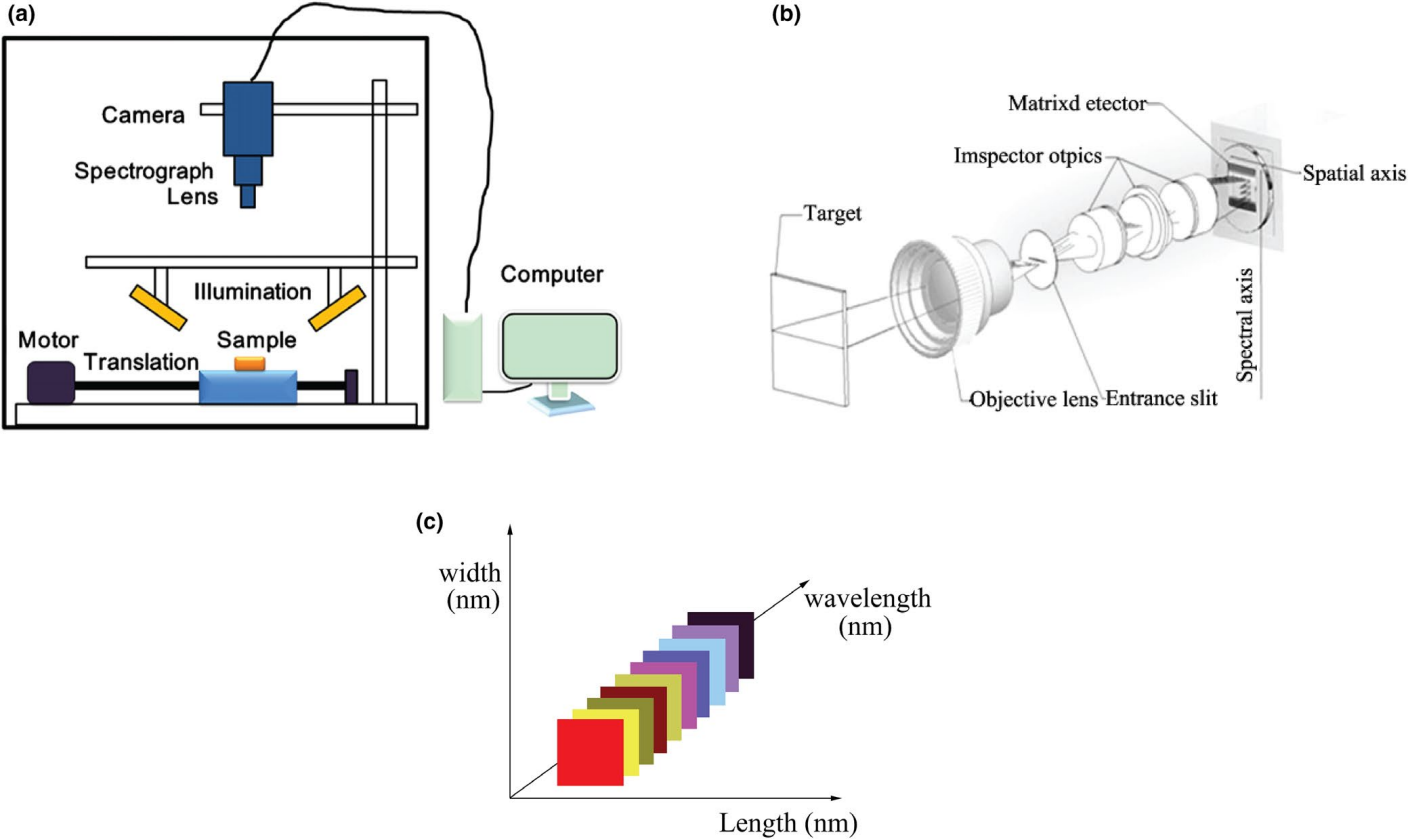
The principle and process of hyperspectral imaging technology. (a) Hyperspectral imaging system (Wu et al., 2012). (b) Diagram of hyperspectral imaging principle (Li & Rao, 2011). (c) Three‐dimension data cube of hyperspectral imaging

The hyperspectral imaging system is mainly consisted of four parts: hyperspectral camera, light source, carrier stage, and computer software and hardware (Figure 1a; Wu et al., 2012). The light emitted by the source is absorbed and then reflected by object surface (Figure 1b; Li & Rao, 2011). After passing through the front lens and entrance slit, light with different wavelengths will have bend‐divergence propagation of different level. Then, it converges at the collimation lens, light of different wavelengths form separate bands by splitting. Finally, the spectral signal will be presented to the detector through the imaging lens. The three‐dimension data cube rich in image and spectral information are obtained by machine sweeping (Figure 1c). Moreover, when choosing the light source, we should pay attention to highlight the object and weaken the background. Meanwhile, to present useful signal as much as possible, the signal‐to‐noise ratio of the image should be improved, thus reduce noise interference (Dong, Guo, Xu, & Xu, 2018). Imaging spectrometer is also called hyperspectral camera, which can absorb, process, and transmit the reflection spectrum of target, is one of the most core part of the whole hyperspectral system. The main function of the electronic control platform is to control the moving speed of the object and make it consistent with the sampling frequency and exposure time of the camera, thus prevent the phenomenon of missing or repeated acquisition. Data acquisition software mainly control the operation of relevant equipment through parameter setting, thus efficiently completing the data acquisition work.

**FIGURE 2:**
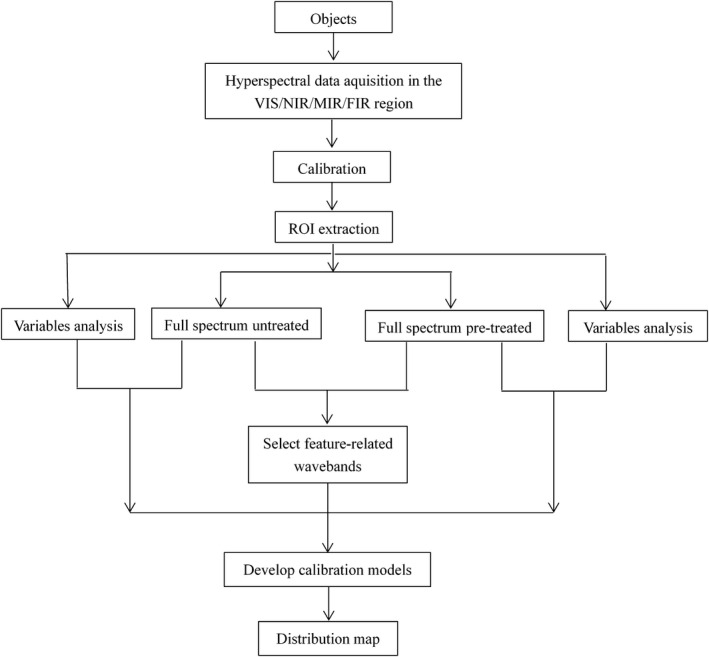
Analysis flow chart of hyperspectral imaging

The hyperspectral off‐line data are processed by chemometrics and computer technology, which is mainly implemented in MATLAB and ENVI software. The general flow chart of data analysis is shown in Figure 2, in which preprocessing, variable selection, and modeling methods are the key steps in the whole analysis process, they all involve a variety of processing algorithms. The selection of algorithms has an important influence on the model accuracy and prediction performance of different variables. The purpose of pretreatment is to remove the noise fluctuation and baseline change generated in the process of data acquisition, so as to enhance the spectral signal. The commonly used spectral pretreatment methods include multiplicative scatter correction (MSC) and standard normal variate (SNV). The spectral information generated in data acquisition originates in the overlap of signals of various chemical substances of sample. The characteristic wavebands closely related to the variables are selected by some methods, which is helpful to improve the predictive effect of the model on variables. The commonly used screening methods include successive projections algorithm (SPA), principal component analysis (PCA), or a combination of various methods, which can further reduce the amount of calculation and improve work efficiency. For different characterization indicators, different models are used for rapid prediction, which is conducive to improving the prediction performance of the model. The commonly used methods of preprocessing, variable selection, and modeling are shown in Table 1.

**TABLE 1:** Statistical tables of commonly used spectral pretreatment, variable selection, and modeling methods

Pretreatment methods	Variable Screening Methods	Modeling methods
Multiplicative scatter correction (MSC); normalized; standard normal variate (SNV); Savitzky‐Golay 1st order (SVG‐1) and 2nd order derivatives (SVG‐2; Crichton et al., 2017)	Successive projections algorithm (SPA); regression coefficient (Caporaso, Whitworth, Grebby, et al., 2018)	Genetic synergy interval partial least square (GA‐Si‐PLS) algorithm (Ling et al., 2017)
Autoscale (Sun et al., 2017)	Competitive adaptive reweighted sampling (Tian et al., 2018)	Partial least squares discrimination analysis (PLS‐DA; Sun et al., 2017)
De‐trending (Caporaso, Whitworth, Grebby, et al., 2018)	Weighted values (Qu et al., 2017)	Partial least square regression (PLSR; Cheng et al., 2018)
	Principal component analysis (PCA; Munera et al., 2017)	Linear and quadratic discriminant analysis (LDA and QDA)；support vector machine (SVM; Munera et al., 2017)
	Genetic synergy interval partial least square (GA‐Si‐PLS) algorithm (Ling et al., 2017)	Artificial neural network (ANN)；multi‐layer perceptron (MLP) neural networks (Orina et al., 2017)
	Two‐wavelength combination method (Xie et al., 2018)	East squares‐support vector regression (LS‐SVR; Wei et al., 2019)
	Random frog (RF) algorithm ‐SPA (Wei et al., 2019)	

## 3 APPLICATION OF HYPERSPECTRAL TECHNOLOGY IN THE FIELD OF AGRICULTURAL PRODUCTS AND FOOD DETECTION

Food detection includes safety and comprehensive detection (Huang, Yao, Hui, Sun, & Xing, 2012). Among them, safety detection refers to the detection of substances that may cause harm to human health in food. Comprehensive detection is also divided into external and internal detection, that is, the detection of external defects and internal quality of objects. Detection is usually carried out by random sampling combined with chemical analysis, which will inevitably prolong the detection time and reduce the accuracy of the result. With the introduction of hyperspectral technology, it has been widely used in the field of food detection (Table 2).

**TABLE 2:** Application of hyperspectral technology in food detection

Plant‐product industry	Animal husbandry	Aquatic farming industry	Others
Maize	Lamb	Cod slices	Nutriments
Brown rice	Chicken	Grass carp fillets	Cocoa beans
Corn	Pork	Shrimp	Coffee bean
Peach	Beef		Nongfu mountain spring
Apple	Milk powder		
Persimmon			
Grape			
Rape			
Mushroom			

### 3.1 Plant‐product industry

Planting industry is the basic sector of the whole agriculture and is also the foundation of human existence, such as grain, cotton, and oil. Plant‐product industry mainly cultivates a variety of crops, according to the property and purpose of products, which can be divided into grain crops, cash crops, feed and manure crops, vegetables, etc.

Grain crop is one of the most important food for human beings, but it is easily infected by fungi during growth and storage, which leads to the decline of the yield and nutritional value (Orina, Manley, & Williams, 2017). In order to identify the situation of food infected by fungi, we can only wait for visible colonies on the surface, or conduct early identification by microbial culture, but this method is time‐consuming and laborious. Williams et al. (Williams, Geladi, Britz, & Manley, 2012) used hyperspectral to detect the changes of fungi on maize surface after infection with *Fusarium verticillioides*. It was found that the fungal change could be identified by hyperspectral technology in the early stage of infection; meanwhile, the content changed significantly after starch and protein were utilized by fungi. However, the infection ability of fungi to different biological samples is different, which will interfere with the establishment of the model, so more systematic research is needed to verify its feasibility. In addition, whether brown rice is infected by fungi during storage can also be detected by hyperspectral technology, and the spectral signal decreases with the increase of fungal colonies (Siripatrawan & Makino, 2015). Once grains are infected by fungi, these fungi usually produce toxins that can cause serious harm to human health, such as aflatoxin in corn (Fiore et al., 2010). At present, traditional methods cannot effectively identify aflatoxin in early stage, yet hyperspectral technology can quickly identify it within 48 hr after artificial inoculation with *Aspergillus flavus*, which may be related to the detection limit of the method. In the early stage, the amount of aflatoxin produced by fungi is low, and the consumption of aflatoxin cannot be avoided by the solution transfer in the traditional detection process, so it cannot be effectively identified by this method. However, hyperspectral detection is realized by capturing the spectral signal reflected from the aflatoxin, which is relatively sensitive. In addition, the data collection does not result in the consumption of aflatoxin. Therefore, hyperspectral technology can quickly identify it.

With the improvement of people's living standards, healthy lifestyle accelerates the consumption of fruits and vegetables. Safety is the foundation and quality is the guarantee, that is, only when the safety problem is solved can the quality be improved. The traditional methods of quality detection cannot preserve the integrity of food even cannot be eaten, resulting in a lot of waste, but hyperspectral technology can solve this problem very well. Therefore, hyperspectral technology has been widely used in the field of nondestructive detection of fruits and vegetables. With the invasion of microbes and their own respiration, the chlorophyll content in the tissue structure gradually decreases, which affects the quality of fruits and vegetables and greatly reduces commodity value. So, Sun et al. (2017) used hyperspectral to detect the content of chlorophyll to reflect the spatial distribution of diseased peach parts. The discovery can provide a new perspective for the identification and classification of fruit quality. In addition to the irreversible effect of decay on the quality of fruits and vegetables, some physicochemical properties also have adverse effects on their taste, nutrition, and shape. Tian, Li, Wang, Fan, & Huang (2018) used hyperspectral technology combined with partial least squares (PLS) model to establish a two‐layer model of soluble solid in apple. It was found that carotenoids have an important impact on the prediction of soluble solid content. Hyperspectral technology is also suitable for rapid prediction of color, quality, and hardness of other fruits (Rajkumar, Wang, Eimasry, Raghavan, & Gariepy, 2012; Xie, Chu, & He, 2018), such as the maturity of persimmon (Munera et al., 2017), anthocyanin in grape (Fernandes et al., 2011), and the difference of trace components in rape under different fertilization conditions (Zhang, Fei, Yong, & Gong, 2013). Hyperspectral technology can also be used to identify the damage of vegetables, so that there is quality assurance before sale, which cannot only ensure the freshness of vegetables in the storage cycle, but also make consumers feel at ease to buy. Mushroom is easy to be damaged in the process of transportation. To solve this problem, Gowen et al. (2008) effectively detected the damage of white mushroom during transportation through hyperspectral technology, and the technology can be used for rapid identification the damage of white mushroom on the production line. Therefore, these studies show that hyperspectral technology can effectively achieve the rapid and nondestructive detection of internal and external quality of fruits and vegetables, so as to ensure the freshness and quality.

### 3.2 Animal husbandry

Meat products are nutritious and delicious, and are very popular with consumers. However, some illegal trader sell unqualified meat for their own huge benefit, which seriously violates the rights and interests of consumers. Kamruzzaman, Elmasry, Sun, & Allen (2011) not only used hyperspectral technology to quickly discriminate the muscle of three different parts of lamb, but also established prediction models for the identification of muscle pH, color, and mass loss (Kamruzzaman, Elmasry, Sun, & Allen, 2012). In order to further reveal the adulteration of meat products, the team (Kamruzzaman, Sun, Elmasry, & Allen, 2013) again used hyperspectral to quickly and effectively detect the content distribution of adulterated ingredients in lamb meat. Chicken is a kind of meat food which is easy to deteriorate even in low temperature. Total volatile basic nitrogen (TVB‐N) is an important indicator to detect the deterioration of chicken. Hyperspectral imaging technology can detect it quickly and nondestructively (Khulal, Zhao, Hu, & Chen, 2016), prevent spoiled chicken from entering the market, and endanger human health. Deep‐frozen can prolong the shelf life of meat, which is a common method for meat storage. However, due to oxidation reaction and mechanical damage, the flavor of meat will decrease seriously during freezing. With carbonyl content as an indicator, the oxidative damage of pork myofibrils during frozen storage can be well measured by hyperspectral technology (Cheng, Sun, Pu, & Wei, 2018). With the pH value, color, and tenderness of beef as indicators, hyperspectral technology can detect the difference in beef with different freshness (Crichton et al., 2017), the water holding capacity of fresh beef (Eimasry, Sun, & Allen, 2012) and accurately classify the beef grade (Elmasry, Sun, & Allen, 2011). In addition, the content of melamine in milk powder can also be effectively determined by hyperspectral technology, so this technology can be used as an effective tool to detect illegal additives in milk powder, thus ensuring the health of consumers (Lim et al., 2016).

### 3.3 Aquatic farming industry

Parasite is major factor that endangers the health and growth of animals in aquaculture. Once the case broke out, it would cause extensive death and heavy loss. Aquatic products have the characteristics of high protein, low fat, and delicious taste, and are easy to become hosts of parasites. If the food contaminated by parasites is not handled properly, it will cause the symptoms of vomiting, diarrhea, etc. Every year, food safety incidents caused by eating parasites emerge in endlessly. However, there has been a lack of effective technology for nondestructive detection of aquatic products that may contain parasites, yet the emergence of hyperspectral technology makes up the deficiency. Based on hyperspectral technology, Sivertsen, Heia, Hindberg, & Godtliebsen (2012) proposed an effective and rapid method to identify nematodes in cod slices, which proved the advantage of hyperspectral detection in meat parasites. This method is suitable for large‐scale detection of aquaculture industry. Meanwhile, hyperspectral technology has also been introduced into the detection of viable count on the surface of fish (Wu & Sun, 2013). For aquatic products, in addition to the detection of parasites and microorganisms, some chemical compounds are also important indicators of internal quality (Elmasry, Sun, & Allen, 2013). Qu, Sun, Cheng, & Pu (2017) established a visual distribution map of the moisture content of grass carp fillets during freeze‐drying by hyperspectral technology. Moreover, they also successfully identified different grades of shrimp using hyperspectral technology (Qu et al., 2015). However, some important information may be lost in spectral preprocessing, and the established model is not universal. Therefore, how to improve the accuracy and prediction performance of the model is one of the important research directions.

### 3.4 Others

Hyperspectral technology has been widely used in the food field. In addition to the primary agricultural products and food mentioned above, this technology has also been applied to other kinds of food detection, such as nutriments (Shi et al., 2017) and cocoa beans (Caporaso, Whitworth, Fowler, & Fisk, 2018). Moreover, hyperspectral technology is first used to quantitatively predict the content of sucrose, caffeine, and triglycerides in single coffee bean (Caporaso, Whitworth, Grebby, & Fisk, 2018). The research of hyperspectral technology is not just a small‐scale validation test, but also has been industrialized. The scientific and technological product line of Nongfu Mountain Spring (a famous drinking water manufacturing enterprise in China) is a typical example of industrialized application of hyperspectral imaging technology. Through hyperspectral photography system combined with computer technology, the types and area of fruit surface defects are identified, and the illumination sorting of fruit is realized. Meanwhile, the near‐infrared detection system form spectral curve by irradiating fruit surface to realize nondestructive detection of sugar and acidity. Before doing this work, it is necessary to establish a very large database, the more databases there are, the higher the accuracy of detection data will be.

## 4 DISCUSSION

Hyperspectral imaging technology has unique advantages in the field of nondestructive testing compared to traditional methods. The traditional detection methods cannot be inseparable from a large number of reagent preparation, instrument use, and manual operation, so the error caused by those factors cannot be estimated. However, for hyperspectral technology, in addition to the basic data used in first modeling needs to be obtained by traditional methods, we only need to extract the hyperspectral data of the sample by taking photographs during the detection, which can be combined with machine learning algorithm to realize the detection of sample quality. Therefore, hyperspectral detection avoids many errors caused by external factors, such as reagents, instruments, and operators, so as to achieve accurate, rapid, and nondestructive detection. However, hyperspectral photography system can only obtain the data information of the object surface, and cannot irradiate the interior of the sample, so it is not suitable for the detection of chemical components with poor homogeneity. In addition, due to the complexity of samples detected, it should be noted that different kinds of samples correspond to different parameter calibration in the process of hyperspectral data acquisition, or the effect of image acquisition and accuracy of data will be affected, thus seriously reduces the credibility of the results.

In the process of data analysis, various mathematical algorithms are commonly used to select characteristic bands, which is actually controversial. Because for the same detection indicator, different algorithms obtain various characteristic bands, so the models established are also different. According to the principle of spectral molecular vibration, all kinds of particles in each substance, such as molecules, atoms, nuclei, and electrons, are moving continuously at a certain energy state. The maintenance of motion needs energy supply, when electromagnetic wave transmits energy to substance, the particles in the substance undergo energy level transition and change from ground state to excitation, the state of motion changes accordingly. However, due to the instability of the particles in the excited state, it releases energy in the form of electromagnetic waves and returns to the ground state, which resulting in complex absorption, reflection, or transmission spectral signal. Therefore, starting from the principle of spectral generation, the characteristic absorption signals of chemical indicators are mainly produced by the vibrations of molecular bonds and functional groups, which are carried by chemical structural formulas of the indicators. In order to improve the robustness and predictive performance of the model, it is suggested that the characteristic wavebands of functional groups of the characterization indexes should be obtained from the relationship between absorption spectrum and molecular structure through gradient experiment design of the pure material, and then, it is brought into the sample to verify the accuracy of the band selection. In addition, it is necessary to prevent other compounds' signals from concealing the spectral information of the detection indicators, which involves spectral decomposition that is a difficult research work. Spectral signals can be optimized through well‐directed experimental design or development of new algorithms.

## 5 CONCLUSION AND PERSPECTIVE

As a image data technology, hyperspectral imaging technology has the advantage of union of imagery and spectrum. It can simultaneously detect the surface and internal information of objects, so as to realize the rapid and nondestructive detection of food quality and safety. Therefore, it has been widely used in the field of food. In this paper, from the perspective of agricultural classification, the research progress of hyperspectral technology in primary agricultural products and food in recent years was systematically reviewed. In addition, the deficiencies and key points of this technology in the research were discussed in depth. This work lays a solid foundation for peers to quickly grasp the application progress of hyperspectral technology in the field of agricultural products and food, contributing to the in‐depth research and application of this technology.

In China, solid‐state brewing (Liu et al., 2004) has a very long history and peculiar culture, and the open fermentation of multi‐strains is the main characteristics of making process. Baijiu and vinegar are typical representatives of solid‐state brewing. Based on hyperspectral technology, the distribution of moisture and acidity in vinegar fermented grains is quickly detected (Zhu et al., 2016), enabling baijiu enterprises to find problems quickly, and adjust processes in time, thus ensure the product quality. It shows that it is feasible to apply hyperspectral imaging technology to the rapid detection of characterization indicators in solid‐state fermentation process. As one of the six distilled spirits in the world (Zhao, Zheng, Song, Sun, & Tian, 2013), chinese baijiu is brewed by open fermentation condition with natural inoculation. Because of its complex fermentation system, it is difficult to effectively monitor the production process. With the arrival of the mechanization and intelligence of chinese baijiu, hyperspectral technology has a broad application prospect in the field of baijiu making, which will have important guiding significance for the transformation and upgrading of traditional technology and intelligent on‐line monitoring of complex fermentation state.

## CONFLICT OF INTEREST

The authors declare that they have no conflicts of interest.

## AUTHOR CONTRIBUTIONS

Min Zhu collected data, drafted the manuscript, and revised it critically. Dan Huang and Xin‐Jun Hu revised the manuscript critically for important intellectual content. Wen‐Hua Tong, Bao‐Lin Han, and Jian‐Ping Tian gathered data. Hui‐Bo Luo contributed to the design of the work. All authors approve the final version of the manuscript and agree to be accountable for all aspects of the work.

## ETHICAL STATEMENTS

This study does not involve any human or animal testing.
